# Shape-shifting and tumor suppression by PLZF

**DOI:** 10.18632/oncotarget.102

**Published:** 2010-05-04

**Authors:** Robin M. Hobbs, Pier Paolo Pandolfi

**Affiliations:** ^1^ Cancer Genetics Program, Beth Israel Deaconess Cancer Center, Departments of Medicine and Pathology, Beth Israel Deaconess Medical Center, Harvard Medical School, Boston, MA 02115, USA.

The promyelocytic leukemia zinc finger (PLZF) transcription factor is directly implicated in tumor suppression although the relevant target genes remain poorly defined. Shi et al. now implicate smooth muscle α-actin and changes in cytoskeletal architecture as key downstream targets of PLZF in opposing cellular transformation.

PLZF is a transcriptional repressor belonging to the POZ-Krüppel (POK) family of transcription factors with critical roles in oncogenesis, development and stem cell maintenance [1-7]. The amino-terminal POZ domain of PLZF recruits transcriptional co-repressors and histone deacetylase (HDAC) activity while carboxy-terminal Krüppel-type zinc fingers mediate sequence-specific binding to gene promoter elements; leading to stable repression of relevant target genes [8, 9]. PLZF was originally identified from its involvement in chromosomal translocations with the RARA gene in cases of t(11;17) acute promyelocytic leukemia (APL) [10]. Expression of the resulting PLZF-RARα and RARα-PLZF fusion proteins drives acute leukemia development by disrupting expression of both RARα and PLZF target genes [5, 11-13]. RARα-PLZF retains DNA binding domains of PLZF but replaces the POZ domain with a transactivating domain from RARα resulting in activation rather than repression of PLZF target genes. Therefore, abrogation of PLZF function and mis-expression of PLZF target genes are thought to be critical for APL development [5]. Consistent with its role in APL, PLZF expression is associated with growth inhibition and cell cycle arrest through its ability to repress expression of a number of growth promoting and proto-oncogenic genes [14-16].

**Fig. 1:**
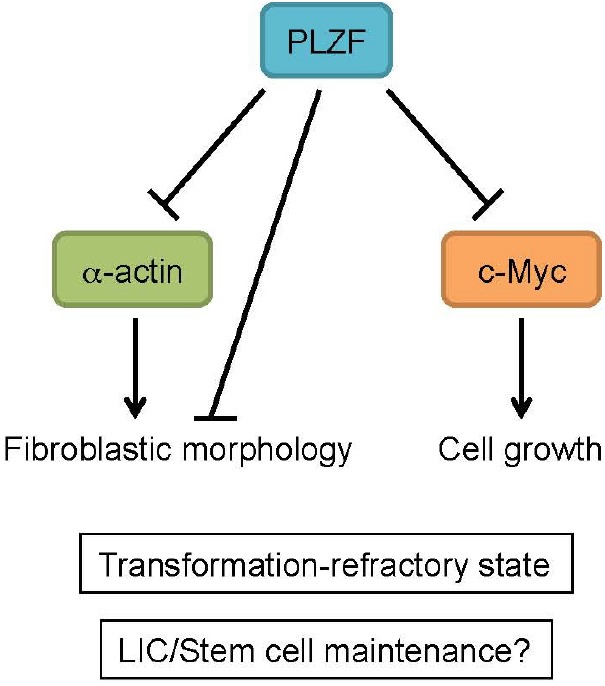
Pathways of Plzf-mediated tumor suppression PLZF can oppose cellular transformation through multiple targets. PLZF expression in CEFs leads to repression of smooth muscle α-actin expression and triggers cells to adopt a flattened, polygonal morphology distinct from the typical fibroblastic shape. This cell morphology is associated with resistance to transformation induced by multiple distinct oncogenes. PLZF also inhibits c-Myc activity by transcriptional and post-translational mechanisms to oppose cell growth and transformation. Regulation of cytoskeletal architecture and c-Myc by PLZF can be relevant to leukemia-initiating cell (LIC) function and stem cell maintenance.

Subsequent to its original characterization in the context of leukemia development, tumor suppressive functions have been attributed to PLZF in other cell types and tissues [17-19]. Using chicken embryonic fibroblasts (CEFs) as a model cell system, Shi et al. have previously demonstrated that PLZF opposes the transformation activity of a variety of cellular and viral oncogenes [20]. In terms of mechanism, the ability of PLZF to inhibit c-Myc function was associated with generation of this transformation-refractory state. In this issue, Shi et al. characterize smooth muscle α-actin as a novel PLZF target gene and link Ras-dependent changes in cytoskeletal architecture to PLZF-mediated inhibition of transformation. This novel mechanism thus represents an additional pathway by which PLZF is able to exert its tumor suppressive function (Figure 1).

Oncogenic stimuli that drive cellular transformation often trigger an accompanying remodeling of the cytoskeleton that results in altered cell morphology and growth properties [21]. Furthermore, the ability of tumor cells to invade surrounding tissue and ultimately metastasize is affected by changes in cell migration involving dynamic alterations to the cytoskeletal network [22]. Shi et al. make the critical observation that expression of PLZF in CEFs induces a reorganization of the actin stress fiber component of the cytoskeleton and alters cell morphology from the typical spindle-shaped fibroblastic shape to a polygonal and flattened one. This morphological change was associated with a direct repression of smooth muscle α-actin expression by PLZF, suggesting that PLZF affects the cytoskeleton through modulation of the levels of specific structural components. Furthermore, expression of dominant negative Ras (RasN17) blocked PLZF-mediated alterations to cell morphology indicating involvement of small GTPases such as Ras, Rac and Rho in this cytoskeletal rearrangement. Importantly, the ability of PLZF to affect cell morphology was linked to its ability to oppose the generation of transformed cell foci by distinct cellular and viral oncogenes; those oncogenes unable to revert the flattened, polygonal phenotype of PLZF-expressing CEFs (e.g. myr-Akt, c-Myc) were successfully opposed by PLZF while those oncogenes that reverted the PLZF-induced cellular morphological changes (e.g. v-Src, v-Jun) did not have their transformation capabilities blocked by PLZF. These results thus define a connection between PLZF-induced changes to cytoskeletal architecture and PLZF tumor suppressor activity while underscoring the importance of cytoskeleton remodeling in oncogene-driven cellular transformation. Taken together, these results offer important insight into the role of PLZF in opposing oncogenesis and raise a number of interesting questions warranting further investigation. Namely, how do PLZF-mediated changes to the actin cytoskeleton inhibit transformation and what are the relevant mechanisms by which certain oncogenes circumvent this? In addition, can this PLZF-driven cytoskeletal remodeling be translated into the context of tumor development where drastic alterations to the cytoskeleton occur, such as during epithelial-to-mesenchymal transition (EMT) and cancer cell invasion? Furthermore, while smooth muscle α-actin is identified as a direct target of PLZF, it remains to be shown whether reduced expression of this gene is entirely responsible for observed changes to the CEF actin cytoskeleton upon PLZF expression. The potential existence of alternative PLZF target genes involved in cytoskeleton remodeling could extend this model of tumor suppression to other cell types where smooth muscle α-actin is not typically expressed. In addition, given the ability of PLZF to oppose c-Myc function at multiple levels in these CEF transformation assays, it will be interesting to assess potential cross-talk between c-Myc, smooth muscle α-actin expression and cellular transformation. Finally, these studies can provide insight and raise new questions regarding the role of PLZF in both leukemia development and stem cell maintenance [2-5, 7]; do the fusion proteins of t(11;17) APL drive leukemogenesis in part, through opposing PLZF-regulated cytoskeletal architecture and does this mechanism of PLZF action have a role in stem cell biology?
